# Loss of MYBBP1A Induces Cancer Stem Cell Activity in Renal Cancer

**DOI:** 10.3390/cancers11020235

**Published:** 2019-02-18

**Authors:** Blanca Felipe-Abrio, Eva Maria Verdugo-Sivianes, Carmen Sáez, Amancio Carnero

**Affiliations:** 1 Instituto de Biomedicina de Sevilla (IBIS), Hospital Universitario Virgen del Rocío (HUVR), Universidad de Sevilla, Consejo Superior de Investigaciones Científicas, 41013 Seville, Spain; bfelipe-ibis@us.es (B.F.-A.); everdugo-ibis@us.es (E.M.V.-S.); csaez@us.es (C.S.); 2 CIBER de Cáncer, Instituto de Salud Carlos III, 28029 Madrid, Spain; 3 Department of Pathology, Hospital Universitario Virgen del Rocío, 41013 Seville, Spain

**Keywords:** MYBBP1A, stem cellness, c-MYB, renal cancer

## Abstract

Tumors are cellular ecosystems where different populations and subpopulations of cells coexist. Among these cells, cancer stem cells (CSCs) are considered to be the origin of the tumor mass, being involved in metastasis and in the resistance to conventional therapies. Furthermore, tumor cells have an enormous plasticity and a phenomenon of de-differentiation of mature tumor cells to CSCs may occur. Therefore, it is essential to identify genetic alterations that cause the de-differentiation of mature tumor cells to CSCs for the future design of therapeutic strategies. In this study, we characterized the role of *MYBBP1A* by experiments in cell lines, xenografts and human tumor samples. We have found that MYBBP1A downregulation increases c-MYB (Avian myeloblastosis viral oncogene homolog) activity, leading to a rise in the stem-like cell population. We identified that the downregulation of MYBBP1A increases tumorigenic properties, in vitro and in vivo, in renal carcinoma cell lines that express high levels of c-MYB exclusively. Moreover, in a cohort of renal tumors, MYBBP1A is downregulated or lost in a significant percentage of tumors correlating with poor patient prognosis and a metastatic tendency. Our data support the role of MYBBP1A as a tumor suppressor by repressing c-MYB, acting as an important regulator of the plasticity of tumor cells.

## 1. Introduction

Tumors are cellular ecosystems where different populations and subpopulations of cells coexist [1]. Tumor cells compete with fibroblasts, vascular and mesenchymal cells, among others for obtaining more resources, more space and proliferate in a Darwinian dynamic. According to the theory of cancer stem cells (CSCs), these are divided into cellular subpopulations of a pyramidal/staggered form of stem cells that give rise to progenitors and these in turn lead to tumor base cells. While the former would be able to regenerate a tumor, the latter would not have this capacity. It has also been proposed that these CSCs would have more resistance to conventional therapies. This is used by numerous therapeutic approaches that seek new drugs to selectively eliminate CSCs. This is based on the idea that the different subpopulations are stable and cannot pass from one state to another, perhaps based on the observation of different species in biological ecosystems. However, tumor cells have an enormous plasticity, are not only metabolic but also phenotypic, and phenomena of de-differentiation of mature tumor cells to cancer initiating cells can occur [2,3]. This tumor evolution can give rise to most resistance to conventional and targeted therapies, and may be responsible for recurrences and residual disease.

A previous loss of function screen to identify genes that might play a role in tumorigenesis [4] identified *MYB binding protein 1A (MYBBP1A)* gene as a potential new tumor suppressor gene. The 160-kDa MYBBP1A, also known as p160, is a nucleolar protein that was originally found to interact with the c-MYB oncogene product. MYBBP1A binds to the leucine zipper motif in the negative regulatory domain (NRD) of c-MYB, being proposed that MYBBP1A could act as a repressor of c-MYB [5]. MYBBP1A also binds to several other transcription factors, such as the PPARγ co-activator 1a (PGC-1a), Prep1 homeodomain transcription factor, the RelA/p65 subunit of NF-kB and p53, playing a pivotal role in its acetylation and accumulation [6,7,8,9,10].

The capacity for which MYBBP1A binds several transcription factors involved in various biological processes, and the fact that MYBBP1A deletion in mice leads to embryonic death prior to blastocyst formation [11], suggest that MYBBP1A is a multifunctional protein involved in several essential biologic processes, such as early embryonic development and cell proliferation. This key role of MYBBP1A, together with the fact that it is located on chromosome 17p13.3, which loses heterozygosity (LOH) at high frequency (up to 50–80%) in many different malignancies, including sporadic breast and ovarian cancer, medulloblastomas, astrocytomas, osteosarcomas, leukemias, bladder, lung, and neuroectodermal tumors [12], could indicate that its main role is to act as a tumor suppressor. However, how MYBBP1A exerts this function remains largely unknown.

In addition, MYBBP1A could be involved in the plasticity of bioenergetics in cancer cells, as MYBBP1A has been proposed to be regulated by the von Hippel-Lindau (VHL) tumor suppressor [13]. pVHL directly binds and degrades MYBBP1A in an iron- and proteasome-dependent manner.

In this work, we characterized the role of MYBBP1A as a new tumor suppressor. We identified that the downregulation of MYBBP1A increases tumorigenic properties due to an increase in stem cell properties probably through c-MYB activation. Interestingly, exclusively renal cancer cell lines that express high levels of c-MYB and do not express pVHL can take advantage of this cellular increase in tumorigenesis. We also analyzed a cohort of renal tumors and found that MYBBP1A is downregulated or lost in a percentage of tumors that show poor prognosis and a metastatic tendency. Our data support the role of MYBBP1A as a tumor suppressor by regulating stemness via repression of c-MYB.

## 2. Results

### 2.1. MYBBP1A Knock Down Increases c-MYB Activity in Renal Carcinoma Cells

The antisense against *MYBBP1A* was found in a loss of function screen to identify new genes involved in tumorigenesis [4], but if the loss of MYBBP1A is an important trait required for the evolution of tumor cells, it must be maintained throughout tumor growth; therefore, we should be able to identify it in human tumors. To confirm this hypothesis, we analyzed the expression of *MYBBP1A* in different types of tumors on cBioportal database and found that clear cell renal cell carcinomas (ccRCC) showed a set of tumors with the lowest expression of *MYBBP1A* (Figure S1). Furthermore, pVHL, which regulates MYBBP1A degradation, is frequently lost in renal cancer; therefore, we decided to use renal tumors and renal carcinoma cell lines as physiological models in our study. 

To explore the potential role of MYBBP1A as a tumor suppressor, we selected 4 different renal carcinoma-derived cell lines (786-O, ACHN, A498 and CaKi-1).

**Figure 1:**
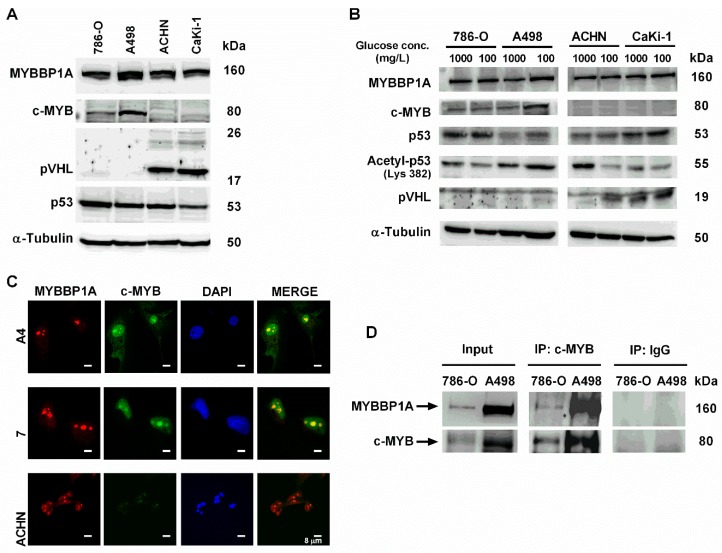
Characterization of MYBBP1A expression and MYBBP1A binding to c-MYB in renal carcinoma cell lines. (**A**) Measurement of MYBBP1A, c-MYB, p53 and pVHL levels in renal carcinoma cell lines A498, 786-O, ACHN and CaKi-1 by western blot. Cells were cultured in 4500 mg/L glucose media. (**B**) Measurement of MYBBP1A, c-MYB, p53, acetyl-p53 and pVHL levels in A498, 786-O, ACHN and CaKi-1 by western blot. Cells were cultured in 1000 mg/L or 100 mg/L glucose media. (**C**) MYBBP1A and c-MYB co-localize in the nucleolus of renal carcinoma cell lines. Cells were stained using DAPI (nuclear control), MYBBP1A and c-MYB antibodies. Scale Bar: 8 µM. (**D**) Co-immunoprecipitation of c-MYB and MYBBP1A in renal carcinoma cells. Protein extracts from 786-O and A498 were subject to immunoprecipitation with c-MYB or normal IgG antibodies. The resultant immunoprecipitates were then analyzed by WB with c-MYB and MYBBP1A antibodies.

It has been proposed that MYBBP1A binds and/or is functionally related mainly to c-MYB, pVHL, and p53 proteins; therefore, we analyzed the levels of these proteins in the selected cell lines. The levels of MYBBP1A were homogeneous in all cell lines and independent on the levels of pVHL (Figure 1A and Figure S2). However, only 2 cell lines, 786-O and A498, expressed clear levels of c-MYB. Interestingly, these 2 cell lines did not express the pVHL protein (Figure 1A). The other 2 cell lines, ACHN and CaKi-1, did not express c-MYB protein but did have high levels of pVHL (mainly isoform 3, which is 19 kDa) (Figure 1A). p53 was mutated in 786-O but retained the WT sequence in CaKi-1, ACHN and A498 [14]. Furthermore, we study the expression of these proteins in diminishing glucose concentrations, as it has been reported that glucose limitation increases MYBBP1A translocation from the nucleolus to the nucleoplasm and binding to p53, enhancing p53 acetylation [15]. However, growing the cells under low glucose conditions did not trigger major variations in these proteins (Figure 1B).

As MYBBP1A was originally found by its interaction with the c-MYB oncoprotein but it remained unclear if MYBBP1A acts as a repressor of c-MYB or not [5], we decided to focus on the effect of MYBBP1A binding to c-MYB. It has been described that MYBBP1A binds to the leucine zipper motif within the NRD of c-MYB, being the c-MYB interacting doming in the N-terminus of MYBBP1A [5] (Figure S3). To explore this relation, we first analyzed whether c-MYB and MYBBP1A co-localized in renal carcinoma cell lines, since c-MYB and MYBBP1A co-immunoprecipitation was previously described but it remained unclear if this interaction takes place in the nucleolus or in the nucleoplasm [5]. After confirming that MYBBP1A was located in the nucleolus of renal cancer cells (Figure S4), as it has been described in other cell types [5,6,11], we observed co-localization of c-MYB and MYBBP1A in the nucleus, specifically nucleoli, of cells with high levels of c-MYB protein (Figure 1C). As we mentioned above, it has been reported that MYBBP1A binds to MYB transcription factor [5]. Therefore, we tested whether MYBBP1A also binds to c-MYB protein in our renal cancer cells by co-immunoprecipitation (Figure 1D). We found that both proteins bound with immunoprecipitation together in 786-O and A498 renal tumor cells, enhancing their possible functional relationship in our model.

**Figure 2:**
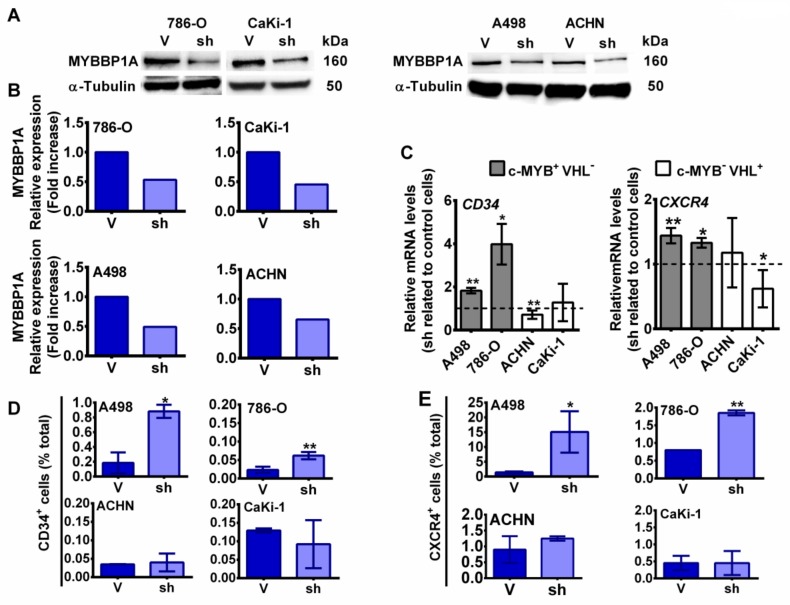
MYBBP1A knock down increases c-MYB activity. (**A**) MYBBP1A knock down by the expression of a specific shRNA. Cell lines were transfected with MYBBP1A shRNA (sh) or a scramble vector (V). After selection, cells were grown to 80% confluence, and proteins were extracted. The figure shows the western blot results of MYBBP1A expression in all cell lines. Scale Bar: 8 µM. (**B**) Quantification of MYBBP1A expression in cells expressing MYBBP1A shRNA (sh) related to the scramble vector (V). (**C**) MYBBP1A knock down increases the expression of c-MYB target genes. mRNA levels of genes directly transcribed by c-MYB were measured by Q-RT-PCR. Graphs represent mRNA levels of cells expressing MYBBP1A shRNA normalized to mRNA levels of control cells. (**D**) Percentage of CD34^+^ cells measured by flow cytometry. (**E**) Percentage of CXCR4^+^ cells measured by flow cytometry. (**B**–**D**) Graphs show the mean ± SD of three independent experiments performed in triplicate. * *p* < 0.05; ** *p* < 0.01.

To explore the effect of MYBBP1A knock down on the 4 renal carcinoma cell lines, we used a shRNA against MYBBP1A and achieved approximately 40–50% reduction of the protein. As negative control, we used a vector that expresses a non-target shRNA (Figure 2A,B). Then, we measured the expression levels of some genes that are directly transcribed by c-MYB activation, such as *CD34* and *CXCR4*, in MYBBP1A knock down cells and in control cells. We found that these genes have increased levels of transcription in A498 or 786-O MYBBP1A knock down cells related to the levels in control cells but not in ACHN or Caki-1 cells (Figure 2C). Similar results were obtained with a second shRNA against *MYBBP1A*. In this case we used an empty vector as negative control, the same vector in which the second shRNA was cloned (V2) (Figure S5).

To confirm the increased expression of c-MYB target genes by MYBBP1A knock down, we measured the percentage of CD34^+^ cells. This is a surface marker not only for genes directly transcribed by c-MYB but also for progenitor hematopoietic, stromal, epithelial and endothelial cells [16]. Again, MYBBP1A knock down in A498 and 786-O cells increased the percentage of CD34^+^ cells (Figure 2D), while this increment was not observed in Caki-1 or ACHN cells. Similar data was observed when we measured the percentage of CXCR4^+^ cells (Figure 2E).

### 2.2. Stemness Capability of MYBBP1A Knock Down Cells

**Figure 3:**
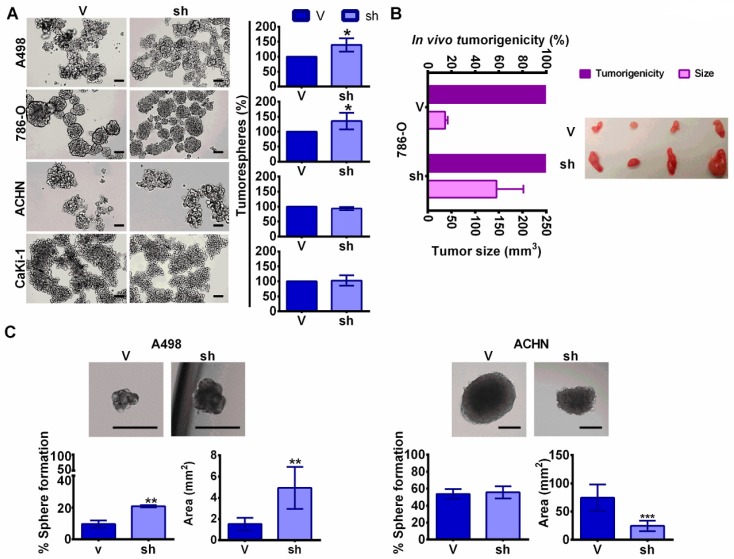
MYBBP1A knock down in c-MYB^+^ cell lines induce cancer stem properties. (**A**) MYBBP1A knock down increases the number of tumorspheres in A498 and 786-O cell lines. Graphs represent the percentage of tumorspheres in MYBBP1A knock down cells compared to control cells. Scale bars: 200 µm. (**B**) Tumorigenicity of tumorspheres in vivo. Tumorspheres from 786-O cells expressing the scramble vector (V) or shMYBBP1A (sh) were injected in nude mice. Graph shows the percentage of tumors formed and the mean ± SD of the tumor size at day 50. *n* = 4. (**C**) Tumorsphere formation from one isolated cell. Graphs show the mean ± SD of the percentage of sphere formation and sphere area. Scale bars: 250 µm * *p* < 0.05; ** *p* < 0.01; *** *p* < 0.001.

Both *CD34* and *CXCR4* are genes directly transcribed by c-MYB that are involved in stem phenotype [16,17], so we decided to study the effect of MYBBP1A knock down in stem properties in our cellular model. Human tumor cell populations can be maintained in serum-free suspension cultures; these populations grow as clusters of cells called “tumorspheres” [18]. These tumorspheres are enriched with multi-potent progenitors [19] with higher expression of CSC markers [20]. We observed that 786-O and A498 MYBBP1A knock down cells formed a higher percentage of tumorspheres than cells expressing the scramble vector (Figure 3A). However, the number of tumorspheres formed by Caki-1 and ACHN were the same in MYBBP1A knock down cells and cells expressing the scramble vector (Figure 3A). Similar results were obtained with a second shRNA against *MYBBP1A* (Figure S6A).

To confirm the tumorigenicity of tumorspheres in vivo, we injected tumorspheres from 786-O MYBBP1A knock down cells and control cells in nude mice. After 50 days, we observed that tumorspheres from both cell lines formed tumors but tumorspheres from MYBBP1A knock down cells formed larger tumors (Figure 3B).

To explore the ability of the cells to form tumorspheres from one single cell, cells were separated by flow cytometry seeding single cells per well in serum-free suspension media. After 1 month, the number and size of tumorspheres formed were measured. We observed increases in the number and size of tumorspheres formed in A498 MYBBP1A knock down cells compared to control cells, while there was no difference in the number of tumorspheres formed in ACHN MYBBP1A knock down cells (Figure 3C).

**Figure 4:**
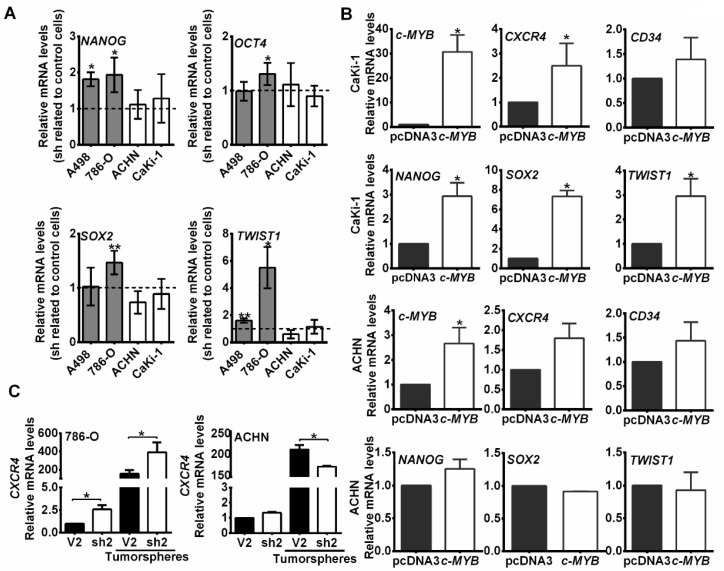
MYBBP1A knock down in c-MYB^+^ cell lines induces the expression of stem genes. (**A**) Measurement of *NANOG, OCT4, SOX2* and *TWIST1* expression by Q-RT-PCR. Graphs represent mRNA levels in MYBBP1A knock down cells normalized to the mRNA levels of control cells. (**B**) CaKi-1 and ACHN cell lines were transfected with *c-MYB* cDNA and the empty vector (pcDNA3). After selection, *c-MYB, CXCR4, CD34, NANOG, SOX2* and *TWIST1* expression were measured by Q-RT-PCR. Graphs represent mRNA levels in cells expressing *c-MYB* cDNA normalized to the mRNA levels of control cells. (**C**) *CXCR4* mRNA levels from adherent cells and tumorspheres were measured by Q-RT-PCR. Graphs represent mRNA levels of 786-O and ACHN cells expressing sh2 normalized to mRNA levels of control cells (V2). (**A**–**C**) Graphs show the mean ± SD of three independent experiments performed in triplicate. * *p* < 0.05; ** *p* < 0.01.

We also measured some transcriptional activators related to the stem cell phenotype (*NANOG, OCT4* or *SOX2*) or to the epithelial-mesenchymal transition (EMT) phenotype (*TWIST1*). We found that *NANOG* and *TWIST1* expression were increased in 786-O and A498 MYBBP1A knock down cells related to the levels in control cells but were not increased in ACHN or Caki-1 cells (Figure 4A). These results were reproduced with a second shRNA against *MYBBP1A* (Figure S6B).

To compare whether the effect of c-MYB overexpression in c-MYB negative cells was similar to MYBBP1A knock down in c-MYB positive cells, we overexpressed *MYB* cDNA in the c-MYB negative CaKi-1 and ACHN cell lines, and measured the *CXCR4, CD34, NANOG, SOX2* and *TWIST* expression. We observed that cells overexpressing c-MYB activate its transcriptional targets in a similar fashion that MYBBP1A knock down cells (Figure 4B).

We also compared the levels of *CXCR4* mRNA levels from adherent cells and tumorspheres by Q-RT-PCR (Figure 4C). We observed an increase in *CXCR4* mRNA levels in 786-O MYBBP1A knock down cells either in the whole population or in tumorspheres only, but this increase was not observed in the ACHN cell line. In addition, we found that the levels of *CXCR4* were much higher in tumorspheres than in the adherent population, as expected from a CSC enriched population.

Finally, we wondered if MYBBP1A knock down altered c-MYB levels, so we measured c-MYB protein levels in our cellular model by western blot. Interestingly, we did not observed significant variations in c-MYB protein levels after MYBBP1A knock down (Figure S7), suggesting that MYBBP1A knock down alters c-MYB target genes expression but does not have a strong effect in c-MYB levels.

Our data clearly showed that MYBBP1A knock down increased the stem cell-like phenotype in some tumor cells and that these tumor cells seemed to have significant levels of c-MYB. On the other hand, cells without c-MYB did not gain selective advantage by MYBBP1A loss.

### 2.3. Tumor Suppressor Phenotype of MYBBP1A in Renal Carcinoma Cells In Vitro and In Vivo 

**Figure 5:**
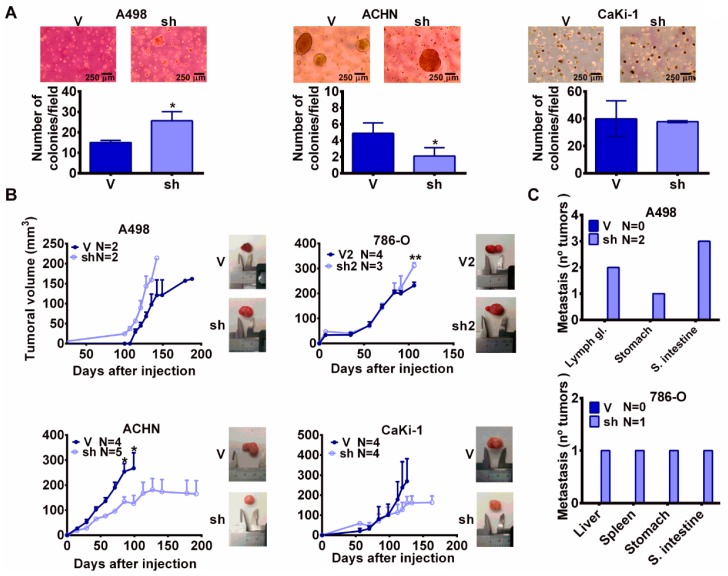
MYBBP1A knock down increases tumorigenicity in c-MYB^+^ cell lines. (**A**) Cells expressing the scramble vector (V) or MYBBP1A shRNA (sh) were seeded in soft agar. After 3-6 weeks, colonies were counted. Scale bars: 100 µm. (**B**) MYBBP1A knock down increases xenograft size of c-MYB^+^ pVHL^−^ cell lines. A498, 786-O, ACHN and Caki-1 control cells (V/V2) or MYBBP1A knock down cells (sh/sh2) were injected in nude mice and tumor size was measured weekly. Graphs represent the tumor size (mean ± SEM). Representative images of tumor size are shown. (**C**) Number of metastasis instances observed in organs of nude mice after injection of A498 and 786-O cells expressing the scramble vector (V) or MYBBP1A shRNA (sh). * *p* < 0.05; ** *p* < 0.0.

The previous data suggest that in our model, MYBBP1A knock down increases stem properties in cells that express high levels of c-MYB, so we wondered if this increase was translated into an increment in tumorigenic properties. To confirm this hypothesis, we performed a soft agar assay and we found that A498 MYBBP1A knock down cells formed more colonies than cells expressing the scramble vector (Figure 5A). The 786-O cells migrated fast, accumulated in the bottom of the plate, and did not form colonies. However, ACHN and CaKi-1 MYBBP1A knock down cells formed the same number of colonies or an even lower number than the control cells (Figure 5A). When these cells were injected in xenografts in nude mice, we observed that A498 and 786-O MYBBP1A knock down cells formed tumors that grew faster than control cells (Figure 5B). However, ACHN and CaKi-1 MYBBP1A knock down cells formed xenograft tumors that were smaller than those of the control cells (Figure 5B). In addition, we observed that A498 and 786-O MYBBP1A knock down cells also metastasized in some host mice before the primary tumors reached large sizes (Figure 5C), while the control cells did not. Then, we analyzed the expression of *MYBBP1A* and EMT-related genes in primary tumors and metastasis from the injected cell lines. We observed that metastasis usually express lower levels of *MYBBP1A*. However, the expression of genes related to EMT, such as *SERPINE1*, *TWIST*, *VIM* or *SNAI1* are increased in metastasis showing an important correlation with MYBBP1A knock down. Interestingly, not all the EMT genes are increased in all metastasis from different organs (Figure S8).

Finally, MYBBP1A knock down cells close the wound in a wound healing biological assay faster than control cells. We observed this difference only in cells that express c-MYB (Figure S9).

These data confirm the tumor suppressor activity of MYBBP1A and points to a connection between MYBBP1A knock down and the induction of a metastatic phenotype in cell lines that express c-MYB (786-O and A498).

### 2.4. MYBBP1A Loss in Human Tumors

**Figure 6:**
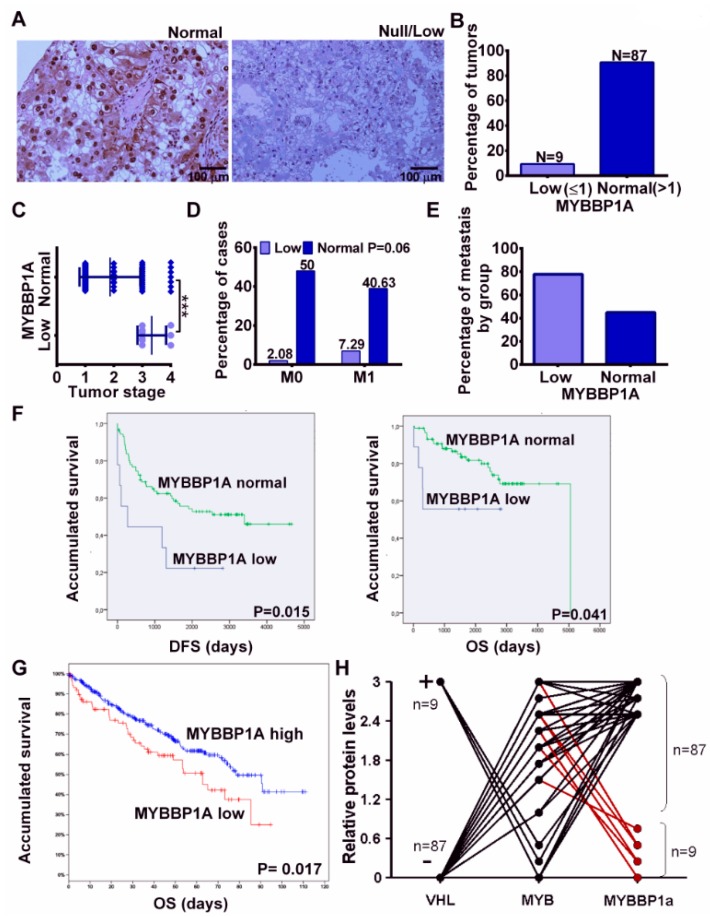
Loss of *MYBBP1A* in human renal tumors. (**A**) Representative images of renal tumors with normal levels of *MYBBP1A* (left) and null/low levels of *MYBBP1A* (right). Scale bar: 100 µM (**B**) Percentage of renal tumors expressing low or normal levels of *MYBBP1A*. (**C**) Correlation of tumor stage with *MYBBP1A* expression levels. *** *p* > 0.001 (**D**) Percentage of cases with metastasis (M1) and without metastasis (M0) in patients with tumors with low and normal levels of *MYBBP1A*. Statistical analysis: Chi-square (**E**) Percentage of relative metastasis in low levels and normal levels of *MYBBP1A* groups. (**F**) Kaplan-Meier curves indicate that patients with tumors with low *MYBBP1A* showed worse disease-free survival and overall survival than patients with tumors with normal levels of *MYBBP1A*. (**G**) Kaplan-Meier curve shows that patients with clear cell renal cell carcinomas (ccRCC) tumors with low *MYBBP1A* showed worse overall survival than patients with tumors with high levels of *MYBBP1A*. Data from TCGA. (**H**) Correlation of c-MYB, MYBBP1A and pVHL expression in renal tumors.

Finally, to study the relevance of MYBBP1A loss in human tumors, we selected a cohort of 96 renal tumors, mostly clear cell carcinomas (Table S1). Renal tumors, as normal renal tissue, show clear staining for nuclear MYBBP1A (Figure 6A). However, in a small number of these cases (approximately 10%), they showed reduced, or even null, expression of MYBBP1A (Figure 6A,B). MYBBP1A loss is not associated with any specific type of renal tumor (Table S2), but showed a clear association with tumor stage (Figure 6C) and metastatic ability of the tumor (Figure 6D,E). Patients with low levels of MYBBP1A protein have worse prognosis for both disease-free survival and overall survival (Figure 6F). In addition, in order to confirm the association of MYBBP1A loss with worse prognosis, we analyzed the overall survival of patients with ccRCC on TCGA database and we observed that patients with tumors with low levels of MYBBP1A showed worse overall survival (Figure 6G).

In sporadic ccRCC, *VHL* is mutated, deleted or epigenetically silenced in around 85–90% of tumors [21]. Therefore, according to our data, the same percentage is expected to maintain c-MYB, given the mutual exclusivity among both genes. Hence, MYBBP1A loss will occur in a tumor with expression of c-MYB and will have functionality by increasing c-MYB activity. To confirm this point we have analyzed the expression of c-MYB in all tumor samples from our cohort and correlated to VHL and MYBBP1A expression. We have also corroborated that all samples with low levels of MYBBP1A contain high levels of c-MYB protein, confirming our hypothesis (Figure 6H).

All these data strongly suggest that the *MYBBP1A* gene is a tumor suppressor and that a loss of this gene favors metastatic phenotypes.

## 3. Discussion

Tumors are complex structures formed by different types of cells. Among these cells, cancer stem cells are considered to be the origin of the tumor mass and the cause of the resistance to conventional therapies. Furthermore, the plasticity of tumor cells allow the de-differentiation of mature tumor cells to cancer stem cells, being essential to identify genetic alterations involved in the regulation of this process to improve the current anti-cancer therapies. We have characterized the role of *MYBBP1A*, a gene identified in a previous screen, in this scenario. We found that the downregulation of MYBBP1A allows cells to survive and proliferate by increasing the CSC phenotype. Our data suggest that MYBBP1A knock down de-represses c-MYB, inducing transcriptional activation of its target genes and an increase in the CSC pool. We believe that the activation of c-MYB by MYBBP1A downregulation confer selective advantages over other tumor cells, leading to clinically relevant renal carcinoma tumors. 

The human *MYBBP1A* gene maps to chromosome 17p13.3 between markers D17S1828 and D17S938. LOH at 17p13.3 is seen at high frequency (up to 50–80%) in many different malignancies [12]. This LOH suggests the presence of one or more tumor suppressor genes at 17p13.3 and *MYBBP1A* could be one of them. It has already been reported that in some contexts, MYBBP1A acts as a tumor suppressor, but no clear mechanism has been given. MYBBP1A downregulation in the NIH3T3 cell line increases cell growth and enhances H-RasV12 tumorigenesis [11]. An opposing function of MYBBP1A in proliferation and migration of head and neck squamous cell carcinoma cells (HNSCC) has also been detected. MYBBP1A downregulation in HSNCC cell lines increases migration but decreases cell growth, generating a sub-population of slow-cycling mobile cells that may be implicated in local tumor recurrence and metastasis [22]. Furthermore, MYBBP1A expression has also been associated with breast cancer tumorigenesis [23]. In this case, MYBBP1A may play a role in tumor prevention by enhancing p53 activation.

MYBBP1A was first identified by its ability to interact specifically with the NRD of c-MYB through leucine zipper-like motifs [5,24]. Thus, it was suggested that MYBBP1A may modulate c-MYB activity upon binding to the c-MYB NRD. Unlike c-MYB, which is expressed mainly in bone marrow, colonic crypt and neurogenic niches [25], MYBBP1A is ubiquitously expressed [12]. We have observed the co-localization of MYBBP1A and c-MYB in the nucleolus of renal carcinoma cells and the co-immunoprecipitation of both proteins, which supports the hypothesis that MYBBP1A modulates c-MYB activity. We have also found that MYBBP1A knock down increases the expression of *CD34*, *NANOG* and *CXCR4*, which are target genes of c-MYB [26,27], exclusively in renal carcinoma cell lines that express high levels of c-MYB. It has been described that c-MYB, NANOG, CD34 and CXCR4 are all involved in the acquisition and maintenance of stem phenotype. c-MYB controls the self-renewal and differentiation of cells, preventing the differentiation to mature cells [25,28,29,30]. NANOG is an essential transcription factor for the maintenance of the pluripotency of embryonic stem cells [31,32,33]. CD34 is a membrane protein originally identified as a marker of hematopoietic stem cells, but there is evidence now establishing CD34 as a general marker of progenitor cells [16]. Lastly, it has been described that CXCR4 is essential for the maintenance of renal CSCs [17]. Although it has not been established a general surface marker for renal CSCs, several studies proposed CXCR4 as a good marker for identifying renal CSCs [34,35,36]. Therefore, we propose that loss of MYBBP1A leads to an increase in c-MYB activity, which controls self-renewal and differentiation of cells and induces the transcription of genes involved in the stem phenotype, thus increasing and potentiating stemness.

We have also found that the downregulation of MYBBP1A increases the tumorigenic properties in vitro and in vivo. Interestingly, the effect of MYBBP1A as a tumor suppressor is only observed in the presence of c-MYB. The deregulation or overexpression of c-MYB has been detected in a wide variety of human cancers and is associated with poorly differentiated tumors, including leukemia, colon and breast tumors. The association of c-MYB with many types of tumors in a diverse set of tissues, suggest that c-MYB plays a critical role in tumorigenesis [26]. However, the mechanisms regulating c-MYB activity and the function of c-MYB in normal and tumor cells is not completely defined [25,26]. Here we show that MYBBP1A acts as a negative regulator of c-MYB, as loss of MYBBP1A leads to an increase of c-MYB activity in renal carcinoma cell lines. In contrast, we could not study the effect of c-MYB silencing in this context as downregulation of *c-MYB* by shRNA fully halts cell growth in our cell lines (Material not intended for publication [37]). On the other hand, we observed exclusivity in the expression of c-MYB and pVHL, but not with MYBBP1A. Although MYBBP1A has been proposed to be regulated by VHL [13], we did not observed this effect and MYBBP1A was not down-regulated upon VHL overexpression (Figure S2). A requirement of both pVHL isoforms 1 and 3 or other renal cancer-specific ubiquitin ligase, as suggested by the observation that MYBBP1A is ubiquitinated in the absence of VHL [13], could explain this discrepancy.

Interestingly, we have observed that the downregulation of MYBBP1A in ACHN cell line reduces the number of colonies and tumorsphere size besides being negative for c-MYB. In this line, it has been detected an opposing function of MYBBP1A in proliferation and migration of head and neck squamous cell carcinoma cells (HNSCC) [22]. It has been reported that silencing of MYBBP1A expression in HNSCC cell lines produces increased migration but decreased cell growth [11]. This may suggests that in some molecular contexts MYBBP1A downregulation may decrease cell growth [10]. 

Finally, we have observed that approximately 10% of renal carcinomas have reduced MYBBP1A expression. Furthermore, we have confirmed that renal tumors with low MYBBP1A levels also show high levels of c-MYB. We also found that loss of MYBBP1A is associated with a metastatic tendency and poorer prognosis in human tumors. The data from our xenograft models also point to this association, but the number of mice where we observed metastasis is low, so further studies will be needed to corroborate this result in mice. Even then, this association supports the fact that the CSC phenotype is the result of MYBBP1A loss, as it has been reported that CSC are involved in metastasis and tumor recurrence [3,38].

## 4. Materials and Methods 

### 4.1. Cell Culture

The ACHN human kidney adenocarcinoma and A498 human kidney carcinoma cell lines were obtained from Cell Line Servic (CLS, Eppelheim, Germany). The 786-O human kidney carcinoma and CaKi-1 human kidney clear cell carcinoma cell lines were kindly provided by Carmen Blanco Aparicio (CNIO, Madrid, Spain). No further authentication of these cell lines was performed by the authors. ACHN and A498 were maintained in DMEM (AQmedia, Sigma, St. Louis, MO, USA). 786-0 and CaKi-1 were maintained in RPMI-1640 (AQmedia, Sigma). All media were supplemented with 10% fetal bovine serum (FBS) (Gibco, Paisley, UK), penicillin, streptomycin and fungizone (Sigma).

### 4.2. Patient Cohort

The cohort of 96 patients for immunohistochemistry studies and the correlation of clinicopathological features were obtained from the biobank of HUVR-IBiS (Sevilla, Spain). All patients provided written informed consent according to the protocol approved by the local ethics committee “Research Ethics Committee of the University Hospitals Virgen Macarena and Virgen del Rocío” (CEI 0309-N-15). All tissue samples and patient information were treated according to the Declaration of Helsinki.

### 4.3. Transfections and Plasmids.

Subconfluent cells were transfected with TransIT-X2 reagent (Mirus, Madison, WI, USA) according to the manufacturer’s instructions. At 48 h, cells were seeded in 10-cm plates with media containing a selection drug (0.5–1 µg/mL puromycin, 50 µg/mL hygromycin B and 400 µg/mL G418). Cells were transfected with the following plasmids: pGene Clip-hygromycin negative control (GGAATCTATTCGATGCATAC) (QUIAGEN, Hilden, Alemania), referred through the text as V; pGeneClip shMYBBP1A (TCCCTGTCACGCCTACTTTCT) (QIAGEN #3336312 KH08420H), referred through the text as sh; pRetrosuper-puro, referred through the text as V2; pRetrosuper shMYBBP1A (GCAGAAGGAGTTCAAGAGACTCCTTCTGCAGCTTGTTCTTTTTGGAA), referred trough the text as sh2; pcDNA3-neomicine; pcDNA3-2xFlag-human-c-MYB; pBabe-puro and HA-VHLwt-pBabe-puro. pcDNA3-2xFlag-human-c-MYB was kindly provided by Shengao Jin and HA-VHLwt-pBabe-puro was a gift from William Kaelin (Addgene plasmid # 19234; http://n2t.net/addgene:19234; RRID: Addgene_19234).

### 4.4. Immunohistochemistry

Tissue microarray was processed for immunostaining as previously described [39]. The samples were incubated with primary antibody, anti-MYBBP1A (Proteintech #14524-AP, Manchester, UK) or anti-cMYB (Abcam #ab117635, Cambridge, UK), for 40 min. We quantified the levels of MYBBP1A and c-MYB by immunostaining using a double-blind observation where we assigned discrete values between 0 (no expression) and 3 (normal MYBBP1A expression or high c-MYB expression). The values were averaged.

### 4.5. Wound-Healing Assays

An artificial “wound” was created using a 10 µL pipette tip on confluent cell monolayer in six-well plates in complete medium. Photographs were taken every 4 h until the wound was closed using an inverted microscope (Olympus IX-71, Tokyo, Japan).

### 4.6. Growth in Soft Agar

To measure the anchorage-independent growth, cells were trypsinized. 1 × 10^5^ cells were suspended in 1.4% agarose D-1 Low EEO (iNtRON Biotechnology, Seoul, Korea) growth medium containing 10% FBS, disposed onto 1 mL of a solidified base of growth medium containing 2.8% agarose. After 24 h, media containing 10% FBS was added to each well of six-well dish and renewed twice weekly. Colonies were scored one month after and all values were determined in triplicate. Photographs were taken with a phase-contrast microscope (Olympus CKX41 with integrated camera Olympus SC30, U-CMAD3, Tokyo, Japan).

### 4.7. Tumorspheres Assay

A total of 5 × 10^3^ cells/mL/well were seeded in Ultralow Attachment Plates containing Mammo Cult^TM^ Basal Medium (Human) (Stem Cell Tech, Vancour, BC, Canada) supplied with 0.48 mg/mL hydrocortisone, 0.2% heparin solution and 10% MammoCultTM Proliferation Supplement (Human). After 4 days, the number of primary tumorspheres were measured using an inverted microscope (Olympus IX-71).

### 4.8. Single-Cell Tumorsphere Assay

Single cells were individually seeded through cell sorting (BD FACS Jazz, Singapore) in 96 well Ultra-low attachment plates containing Mammo Cult^TM^ Basal medium (Human) (Stem Cell Tech) supplied with hydrocortisone 0.48 mg/mL, Heparin solution 0.2% and 10% Mammo Cult™ Proliferation Supplement (Human). After 30 days, primary tumorspheres formed were measured both in size and number using inverted microscope (Olympus IX-71).

### 4.9. Fluorescence-Activated Cell Sorting (FACS) Analysis

FACS analysis was performed as previously described by [40]. Fluorochrome-conjugated monoclonal antibodies against human CD34 (FITC, MACS Miltenyi Biotec #130-098-142, Bergisch Gladbach, Germany) and CXCR4 (PE, MACS Miltenyi Biotec #130-098-354) were used.

### 4.10. Xenograft in Nude Mice

Tumorigenicity was assayed by the subcutaneous injection of 5 × 10^6^ cells of 786-O and 10^6^ of ACHN, A498 and CaKi-1 cell lines into the right flanks of 4-week-old female athymic nude mice. Tumorsphere tumorigenicity was measured by seeding 10^4^ cells as described in tumorspheres assay section, after 5 days tumorspheres were disaggregated with 0.025% trypsin-EDTA, resuspended in 50 µL of media and 50 µL of matrigel (Corning, Corning, NY, USA) and injected into the right flanks of 4-week-old female athymic nude mice. Animals were examined weekly. Tumor volume (mm^3^) was measured using calipers. All animal experiments were performed according to the experimental protocol approved by the IBIS and HUVR Institutional Animal Care and Use Committee (0309-N-15).

### 4.11. Q-RT–PCR

Total RNA purification, reverse transcription and qPCR were performed as previously described by [41]. We used the following probes (Applied biosystems, Foster City, CA, USA): β-Actin (Hs0160665_g1), MYBBP1A (Hs00959671_m1), NANOG (Hs04260366_g1), OCT4 (Hs00999632_g1), SOX2 (Hs01053049_s1), TWIST1 (Hs01675818_s1), CD34 (Hs00990732_m1), CXCR4 (Hs00607978_s1), MYB (Hs00920556_m1), SNAI1 (Hs00195591_m1), VIM (Hs00958111_m1) and SERPINE1 (Hs00167155_m1).

### 4.12. Protein Isolation and Western Blot Analysis

Western blots were performed as previously described elsewhere. Membranes were incubated with the following primary antibodies: anti-MYBBP1A (Proteintech #14524-AP), anti-c-MYB (Millipore #05-175, Billerica, MA, USA), anti-pVHL (Santa Cruz # sc-5575, Santa Cruz, CA, USA), anti-p53 (Santa Cruz #sc-6243), anti-acetyl-p53 (Lys 382) (Cell Signaling #2525, Danvers, MA, USA). a-Tubulin (1:10,000, Sigma #T9026) was used as a loading control. Horseradish peroxidase-labeled rabbit anti-mouse (Abcam # ab 97046) and goat anti-rabbit (Abcam # ab 97051) secondary antibodies were used. The proteins were detected using an ECL detection system (Amersham Biosciences, GE Healthcare, Buckinghamshire, UK) and Bio-Rad ChemiDoc XRST (Hercules, CA, USA).

### 4.13. Co-Immunoprecipitation Assays

40 µL of protein A Sepharose (GE Healthcare, Buckinghamshire, UK) were washed twice with PBS-BSA (5 mg/mL) supplemented with a cocktail of protease and phosphatase inhibitors and then incubated with anti-c-MYB (1:250, Millipore #05-175) or anti-IgG (Santa cruz # sc-2025) in the same buffer for 3 h at 4 °C. After 2 washes in PBS-BSA, 1mg of cell extracts were added and incubated overnight. Immunoprecipitates were washed once with PBS-BSA and twice with IGEPAL 0.2%. Proteins were eluted in 40 µL of 5× Laemmli buffer (10% SDS, 25% 2-mercaptoethanol, 50% glycerol, 0.01% bromophenol blue and 0.3 M Tris-Hcl pH 6.8), boiled 5 min and separated by 6% SDS-PAGE.

### 4.14. Co-Localization Assays

Cells were seeded onto glass coverslips in media with 1000 mg/L of glucose, fixed with 4% paraformaldehyde for 20 min and permeabilized with 0.5% Triton X-100 for 5 min. The coverslips were incubated with blocking solution (PBS + 0.1% Triton X-100 + 3% BSA) for 1 h and then incubated for 6–7 h with anti-MYBBP1A antibody (1:50, Abcam #ab89121). The coverslips were washed with PBS + 0.1% Triton X-100 and incubated for 6-7 h with the second antibody, anti-c-MYB (1:100, Abcam #ab117635) or anti-UBF (1:50, Novus biological #NBP1-82545, Centennial, CO, USA). Secondary antibodies anti-mouse Alexa Fluor 546 (1:250, Thermo Fisher #A21123, Eugene, OR, USA) and anti-rabbit Alexa Fluor 488 (1:250, Thermo Fisher #A11008) were used. The nuclei were stained with DAPI, and the coverslips were mounted with Prolong Gold Antifade (Life Technologies, Carlsbad, CA, USA). A confocal ultraspectral microscope (Leica TCS-SP2-AOBS-UV, Wetzlar, Germany) that allowed sequential scanning of emission channels was used for image detection.

### 4.15. Statistical Analyses

Patient cohort was analyzed using the IBM SPSS Statistics package (22.0 for Windows). The distribution of quantitative variables among different study groups was assessed using the parametric Student’s *t*-test. The Kaplan-Meier method was used for survival analysis, using the Cox proportional hazards model to adjust for the explanatory variables and obtain the *p*-values. In addition, the log-rank test was used to compare the survival distributions between groups with normal and low MYBBP1A levels. Disease-free survival (DFS) was defined as the length of time from the date of diagnosis until the date of relapse or progression event. Overall survival (OS) was defined as the length of time from the date of diagnosis until the date of the last medical record. Statistical analyses of experiments were performed using GraphPad Prism (6.01 for Windows). Control samples and MYBBP1A shRNA samples were compared using the unpaired Student’s *t*-test or Student’s *t*-test with Welch’s correction, as appropriate. Experiments were performed a minimum of three times independently and always performed in triplicate samples.

## 5. Conclusions

Our work supports the theory that MYBBP1A acts as a tumor suppressor that interacts with c-MYB. MYBBP1A knock down in renal carcinoma cell lines results into an increase in c-MYB target genes expression, leading to an increase in the stem population. Interestingly, cancer cells that express c-MYB and do not express pVHL are the ones that can take advantage of the MYBBP1A knock down. Finally, loss of MYBBP1A is observed in a significant percentage of patients with renal cell carcinoma, who show a metastatic tendency and poor prognosis, probably due to the increased stem phenotype caused by c-MYB activation.

## Supplementary Materials

The following are available online at http://www.mdpi.com/2072-6694/11/2/235/s1, Figure S1: MYBBP1A expression in human tumors, Figure S2: VHL overexpression does not affect MYBBP1A levels in 786-O cell line, Figure S3: Schematic representation of structural features of MYBBP1A, Figure S4: MYBBP1A is located in the nucleolus of renal carcinoma cell lines, Figure S5: Effect of second shRNA against *MYBBP1A* in genes directly transcribed by c-MYB, Figure S6: Effect of second shRNA against *MYBBP1A* in CSC phenotype, Figure S7: Effect of MYBBP1A knock down in c-MYB levels, Figure S8: Expression of *MYBBP1A* and EMT-related genes in primary tumors and metastasis., Figure S9: Downregulation of MYBBP1A increases migration in c-MYB^+^ cells. Table S1: Patient clinical characteristics of the cohort used in our study, Table S2: Percentage of cases of each renal cell carcinoma subtype in low MYBBP1A and normal MYBBP1A groups.
