# Supplementary material for: Loss of MYBBP1A Induces Cancer Stem Cell Activity in Renal Cancer
Source: Cancers (Basel). 2019 Feb 18;11(2):235. doi: 10.3390/cancers11020235 (PMC6406377; doi:10.3390/cancers11020235)
Supplement: Supplementary file 1 [file cancers-11-00235-s001.pdf]

Supplementary Materials: Loss of MYBBP1A Induces Cancer Stem Cell Activity in Renal Cancer

Blanca Felipe-Abrio, Eva Maria Verdugo-Sivianes, Carmen Sáez and Amancio Carnero

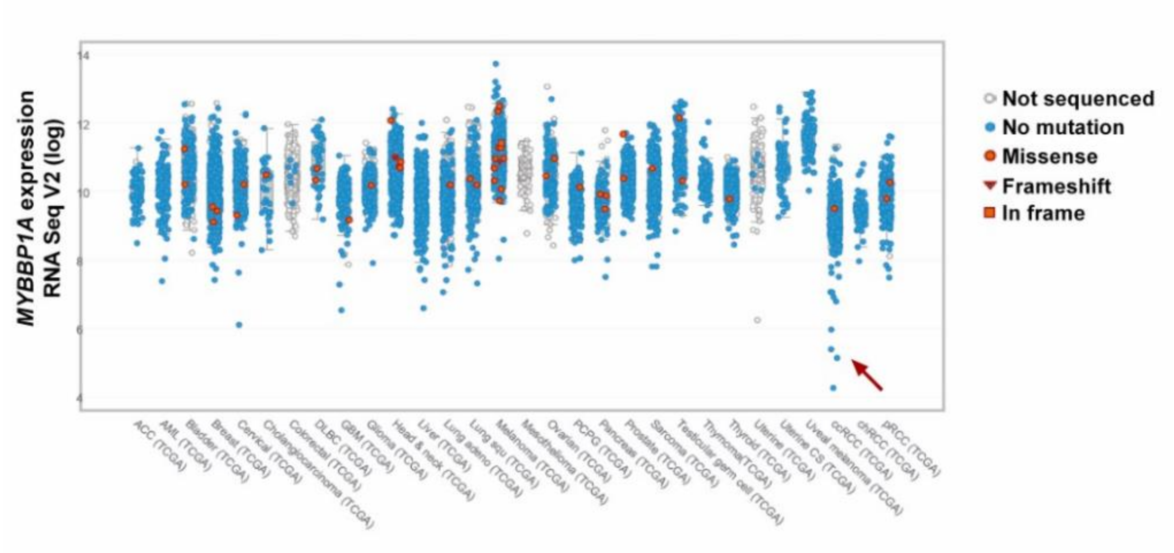

Figure S1. *MYBBP1A* expression in human tumors. Data from cBioPortal for Cancer Genomics. Arrow points ccRCC tumors with the lowest levels of *MYBBP1A* expression.

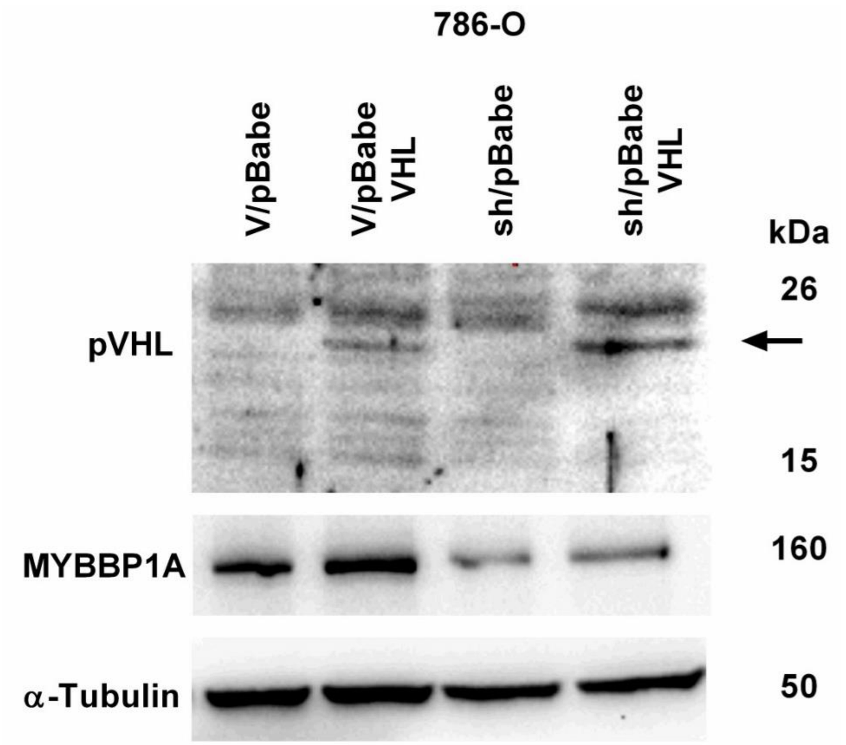

Figure S2. *VHL* overexpression does not affect MYBBP1A levels in 786-O cell line. We overexpressed *VHL* gene in 786-O cells transfected either with scramble vector or sh*MYBBP1A* and we measured pVHL and MYBBP1A levels by western blot.

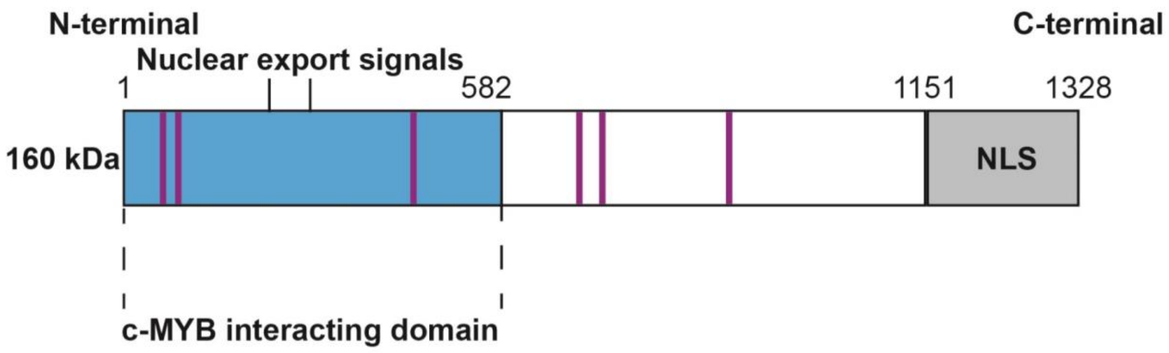

Figure S3. Schematic representation of structural features of MYBBP1A. The domain reported to interact with c-MYB (aa 1-582) is shown in blue. Nuclear and nucleolar localization signal (NLS) domain is indicated in grey. Putative leucine zipper motifs that are conserved between species [1] are shown in purple.

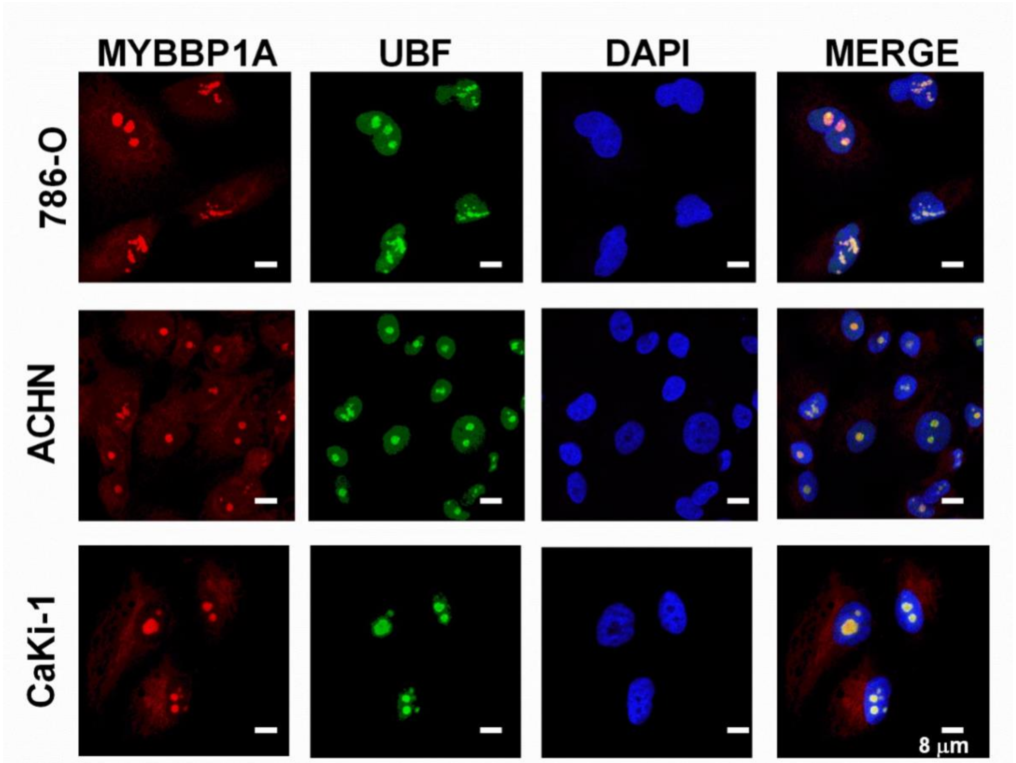

Figure S4. MYBBP1A is located in the nucleolus of renal carcinoma cell lines. 786-O, ACHN and CaKi-1 cells were fixed and stained using UBF antibody (nucleolar control), MYBBP1A antibody and DAPI (nuclear control).

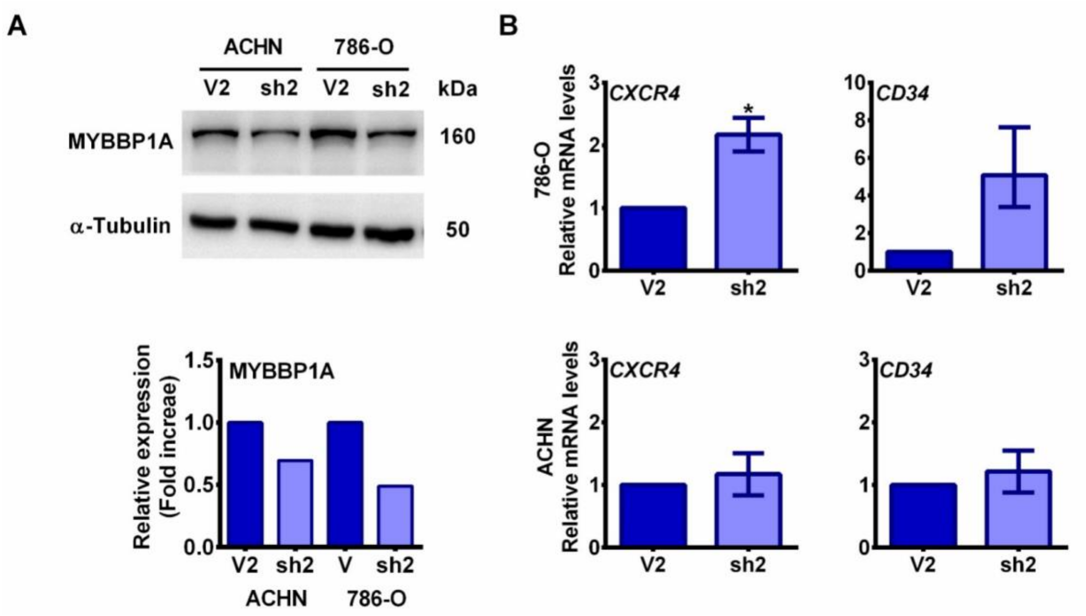

Figure S5. Effect of second shRNA against *MYBBP1A* in genes directly transcribed by c-MYB. (**A**) Validation of MYBBP1A knock down. 786-O and ACHN cell lines were transfected with *MYBBP1A* shRNA2 (sh2) and an empty vector (V2). After selection, proteins were extracted when cells reached 80% confluence and MYBBP1A levels were measured by WB. (**B**) Measurement of mRNA levels of genes directly transcribed by c-MYB in 786-O and ACHN cells expressing *MYBBP1A* shRNA2 (sh2) or the empty vector (V2) by Q-RT-PCR.

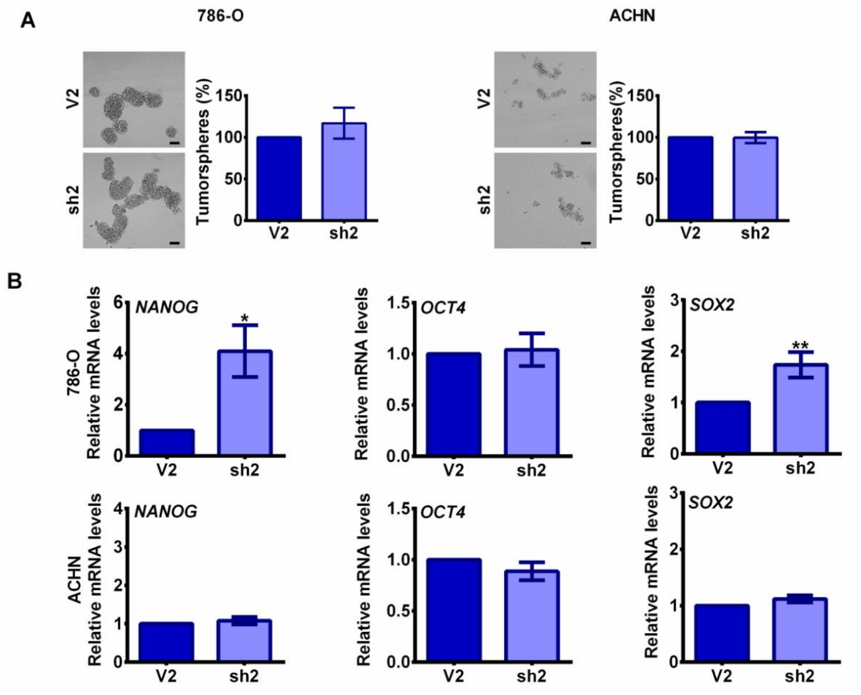

Figure S6. Effect of second shRNA against *MYBBP1A* in CSC phenotype**.** (**A**) Tumorsphere formation assay of 786-O and ACHN cells expressing *MYBBP1A* shRNA2 (sh2) and the empty vector (V2). Scale bars: 200 μm. (**B**) Measurement of mRNA levels of stem genes (*NANOG, OCT* and *SOX2*) by Q-RT-PCR in 786-O and ACHN cells expressing *MYBBP1A* shRNA2 (sh2) and the empty vector (V2).

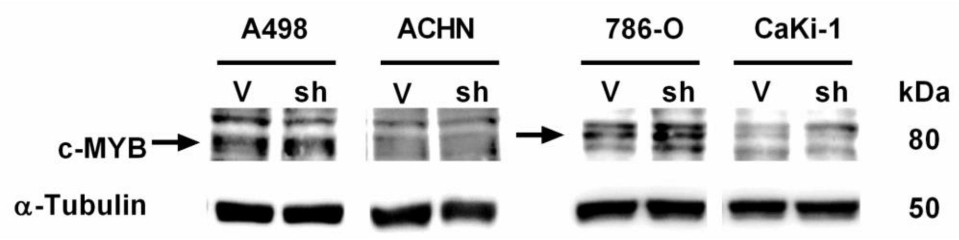

Figure S7. Effect of MYBBP1A knock down in c-MYB levels. Proteins from control and MYBBP1A knock down cells were extracted and c-MYB levels were measured by western blot.

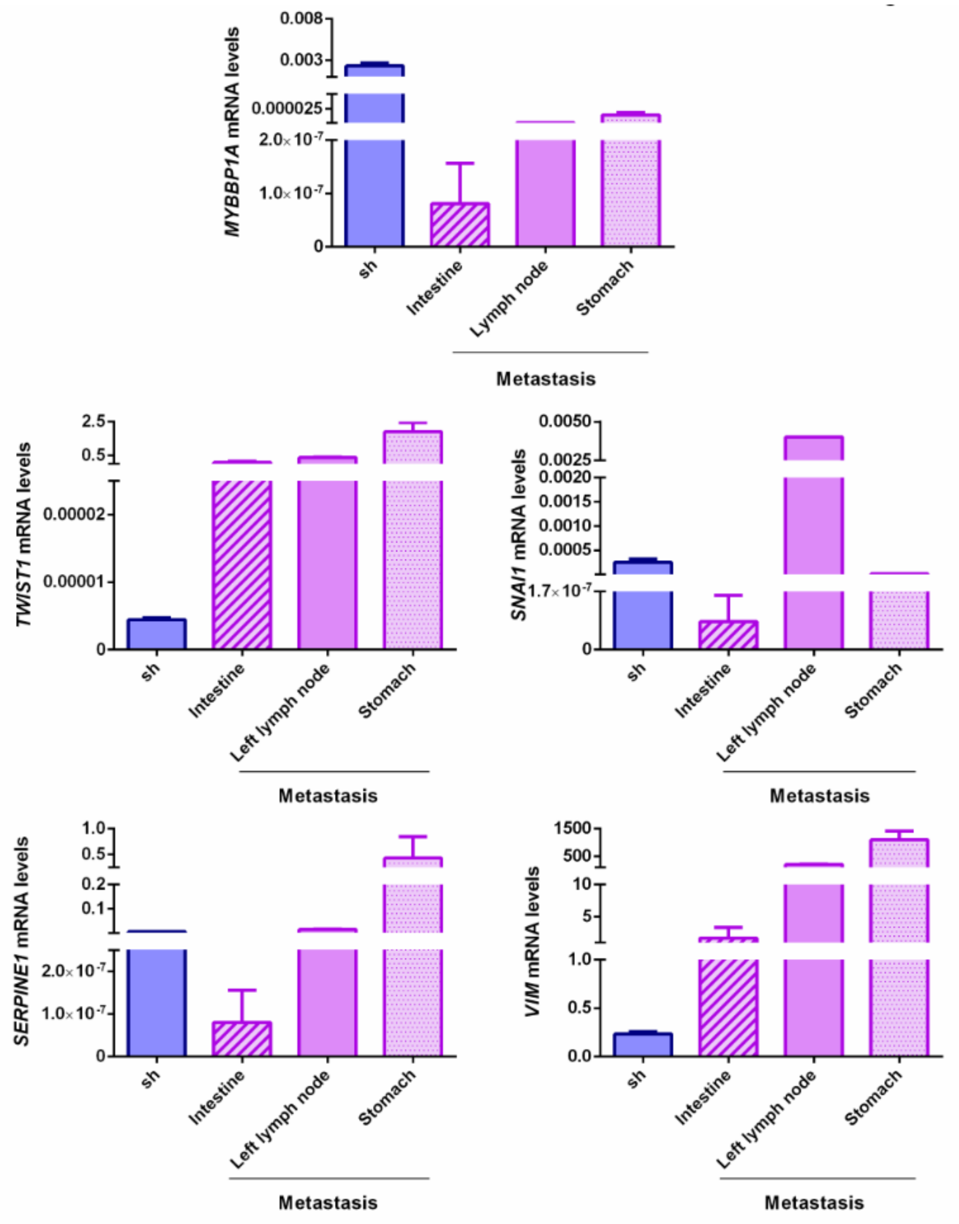

Figure S8. Expression of *MYBBP1A* and EMT-related genes in primary tumors and metastasis. Measurement of mRNA levels of *MYBBP1A*, *TWIST1*, *SNAI1*, *SERPINE1* and *VIM* in primary tumors and metastasis from A498 MYBBP1A knock down cells by Q-RT-PCR.

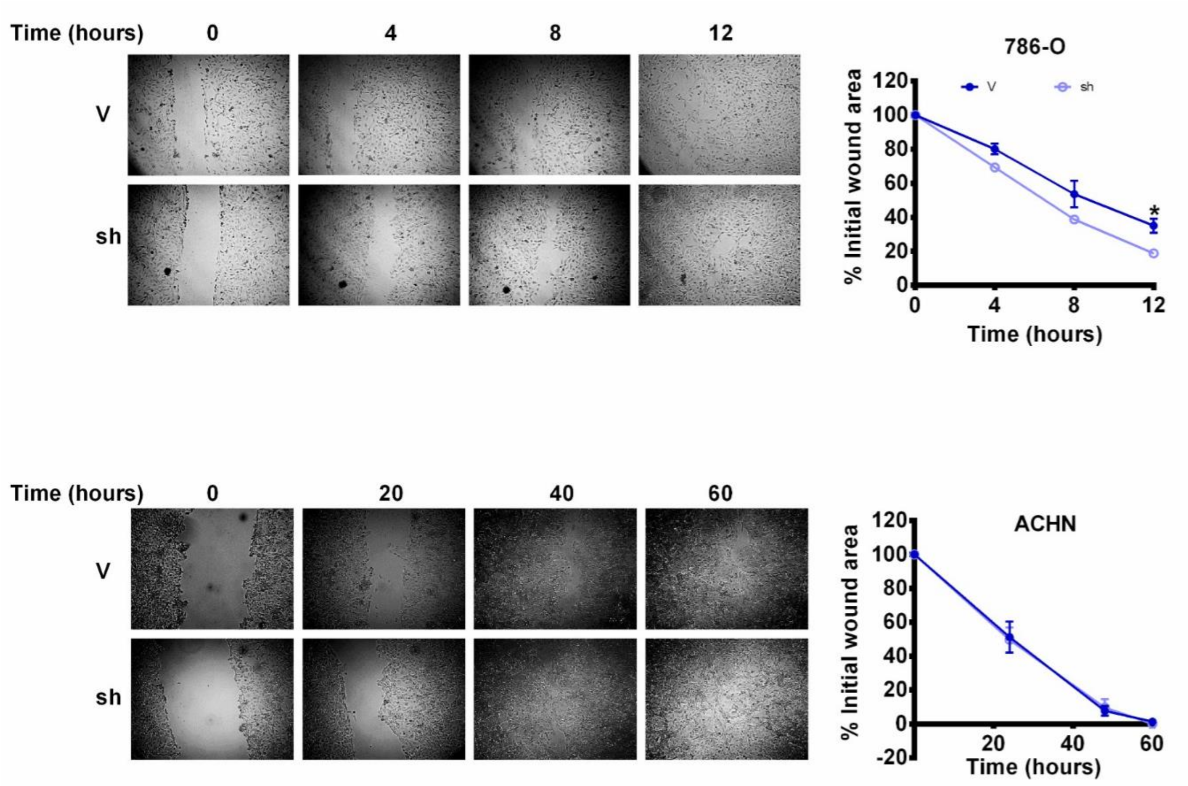

Figure S9. MYBBP1A knock down increases migration in c-MYB⁺ cells. Cell migration was measured by wound-healing assay. Graphs show the mean ±SD of 3 independent experiments performed in triplicate. * $p < 0.05$.

Table S1. Patient clinical characteristics of the cohort used in our study. Related to figure 6.

Population characteristics	Number of cases	Percentage (%)
Normal samples	5	5.21
Type of renal cell cancer (RCC)		
Clear cell (ccRCC)	65	67.71
Papillary cell (pRCC)	19	19.79
Chromophobe (chRCC)	2	2.08
Mixed RCC	3	3.13
Sarcomatoid clear cell RCC	5	5.21
Sarcomatoid cromophobe RCC	1	1.04
Unclassifiable	1	1.04
TNM staging		
I	41	42.71
II	10	10.42
III	23	23.96
IV	10	10.42
Undetermined	12	12.5
Treatment approach		
Surgery	96	100
Radiotherapy	10	10.42
Chemotherapy	48	50
Tk inhibitors	64	66.67
mTOR inhibitors	24	25
Interleukin 2	1	1.04
Capacitabine	1	1.04
Palliative	5	5.21

Table S2. Percentage of cases of each renal cell carcinoma subtype in low MYBBP1A and normal MYBBP1A groups. Related to figure 6.

Type of renal cell cancer (RCC)	Total percentage		Relative percentage by group	
	Low MYBBP1A	Normal MYBBP1A	Low MYBBP1A	Normal SMYBBP1A
Clear cell (ccRCC)	8.33	59.38	88.89	65.51
Papillary cell (pRCC)		19.79		21.64
Chromophobe (chRCC)		2.08		2.30
Mixed RCC		3.13		3.45
Sarcomatoid clear cell RCC	1.04	4.17		4.60
Sarcomatoid cromophobe RCC		1.04		1.15
Unclassifiable		1.04	11.11	1.15

Reference

1. Keough, R.; Woollatt, E.; Crawford, J.; Sutherland, G.R.; Plummer, S.; Casey, G.; Gonda, T.J. Molecular cloning and chromosomal mapping of the human homologue of MYB binding protein (P160) 1A (MYBBP1A) to 17p13.3. *Genomics* **1999**, *62*, 483–489, doi:10.1006/geno.1999.6035.

© 2019 by the authors. Licensee MDPI, Basel, Switzerland. This article is an open access article distributed under the terms and conditions of the Creative Commons Attribution (CC BY) license (http://creativecommons.org/licenses/by/4.0/).
